# Diabetes Mellitus as Hub for Tuberculosis Infection: A Snapshot

**DOI:** 10.1155/2016/5981574

**Published:** 2016-10-12

**Authors:** Rahul Pal, Moiz A. Ansari, Saif Hameed, Zeeshan Fatima

**Affiliations:** Amity Institute of Biotechnology, Amity University Haryana, Gurgaon, Manesar 122413, India

## Abstract

Tuberculosis (TB) still remains the thorn in the flesh of efficient therapeutics affecting one-third of global population annually. There are several factors that enhance the susceptibility to TB infections including malnutrition, smoking, and immunocompromised conditions such as AIDS. In the recent years, growing body of evidence has gained considerable prominence which suggests that Diabetes Mellitus (DM) is individual risk factor leading to complicated TB infections. In this article the authors have attempted to summarize the link of type 2 DM with TB, the mechanistic action of how DM sensitizes for developing the active TB infection from the latent infection, and problems faced during treatment followed by possible preventive measures. We have tried to give account of the alterations that occurred in DM making a person more prone to develop TB.

## 1. Introduction

**Figure 1:**
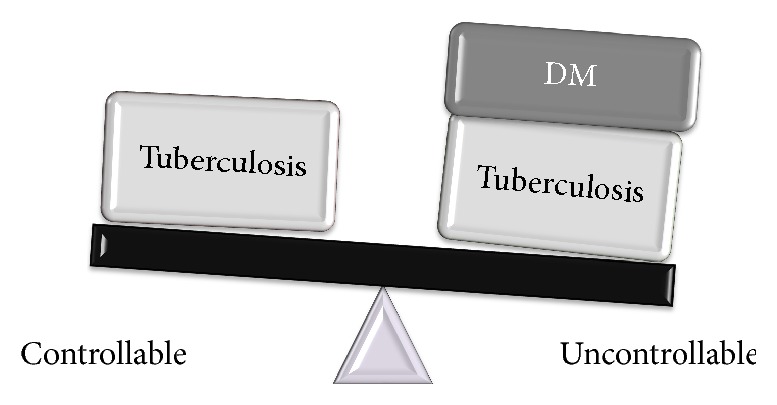
Twin burden of TB infection and DM: TB infections are controllable in comparison to diabetics suffering from TB infection.

Tuberculosis (TB) continues to be a considerable worldwide health issue, provoking millions of new cases and one-third deaths annually. Around 2 billion of the population of the world are considered to be infected with latent TB and out of which approximate 5–10% grows in to active TB [1, 2]. Previously it was thought that TB emerges only in low economic countries but in recent years it is spreading its tentacles to high income countries as well. This shift in the scenario is mainly because of predisposing factors like malnutrition, smoking, and immunocompromised conditions such as AIDS [3–6]. Among various challenging conditions Diabetes Mellitus (DM) is emerging out to be one of the leading factors after HIV/AIDS [7–9]. Epidemiological reports have proved a strong link between DM and TB [10]. DM is a condition developed due to either the pancreas not producing enough insulin (type 1) or the cells of the body not responding enough to the insulin produced (type 2). Type 1 DM was previously referred to as “Insulin-Dependent Diabetes Mellitus” (IDDM) or “juvenile diabetes.” Type 2 DM begins with insulin resistance, a condition in which cells fail to respond to insulin properly and as the disease progresses a lack of insulin may also develop. This form was previously referred to as “Non-Insulin-Dependent Diabetes Mellitus” (NIDDM) or “adult-onset diabetes.” Type 2 DM is more prevalent than type 1 DM and occurs in 90% of the cases. The pervasiveness of DM growing worldwide is predicted to attain an approximate total of 300 million people by 2025. It is believed that DM patients are thrice more susceptible to develop TB as compared to normal individuals without DM. Thus addressing the problem of DM in achieving the long term goal of curing TB is also needed. Various case studies revealed unsuccessful TB treatment outcome in DM-TB patients (Figure 1) as compared to only TB patients [11–13]. Considering the impact of DM on development and treatment of TB, this article on a common platform focuses on the link of type 2 DM with TB, the mechanistic action of how DM sensitizes for developing the active TB infection from the latent infection, and problems faced during treatment followed by possible preventive measures.

## 2. Predisposing Factors Endorsing TB Infection in DM

### 2.1. Latent to Active Transition

**Figure 2:**
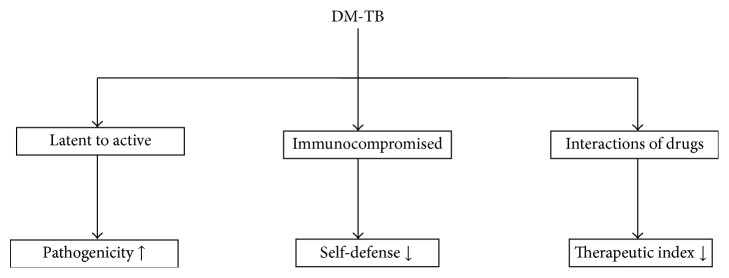
Predisposing factors endorsing TB in DM: DM leads to frequent development of latent to active TB having enhanced pathogenicity; compromised immunity causing loss in self-defense and the interaction of anti-TB drug with antidiabetic drugs causing decreased therapeutic index.

TB is an airborne disease that is transferred from one person to another either through aerosol formation or through contaminated air within the population. However based on the PPD test results survey, usually, only 30% of the population gets successfully infected when coming in contact with MTB. In remaining 70% of the population the boosted level of immunity prevents the onset of MTB infection and prolongs the latent condition. Under this condition most MTB are distorted and the few viable ones are transferred to the latent state because of their hampered metabolism. TB and DM both interact with each other on various levels, one making worse situation for the other [14]. Under diabetic conditions the possibility of death with TB enhances and moreover if it is cured, the chances of reappearance of this infection are high under diabetic condition (Figure 2). Literature also shows that people suffering from DM stay infected more than people who do not have DM. Additionally, the level of blood sugar is elevated temporarily beyond normal in TB; this condition is termed as “impaired glucose tolerance,” which makes a chance for developing DM, and this condition is also called “prediabetes,” a state of hyperglycemia [15–17].

### 2.2. Immunocompromised

**Figure 3:**
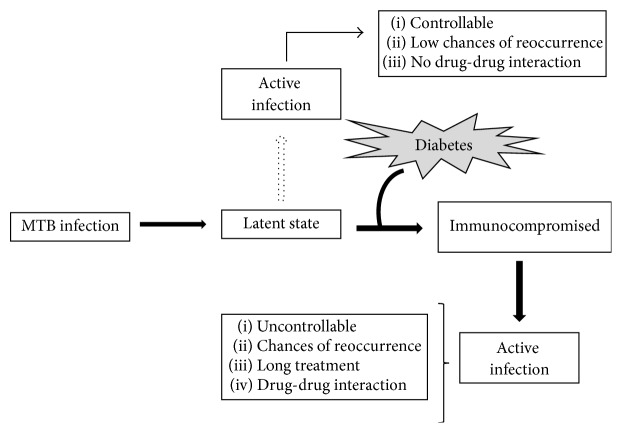
Implications of DM on TB: MTB resides as latent condition which could develop to active TB (shown by dotted arrow) but still is curable. Under diabetic condition immunocompromised state develops making condition prone to develop active TB (shown by bold arrows) which becomes incurable.

To attain the minimal level of defense against MTB, body develops the immunologic responses in the form of first-line defense through innate immunity where eradication occurs through phagocytosis. However, because of the increased burden of MTB the innate immunity further develops the adaptive immunity leading to the activation of localized T-helper 1 (Th1) along with several cytokines (TNF-*α*, IL-1*β*, and IL-12), lymphocytes, monocytes, natural killer T cells, and B lymphocytes that form granulomas and kill almost all MTB preventing the growth of those that remain viable. However in the diabetic conditions the immunologic responses weaken making the individual immunocompromised (Figure 3). In recent in vitro study on diabetic cells the reduced level of MTB phagocytosis was observed in the diabetic monocytes probably due to alteration in diabetic monocytes and C3 component of complement system mainly responsible for MTB phagocytosis [18]. Further, the study on 2-week infected mice with DM leads to reduced uptake of MTB through macrophages which clearly depict the delayed or loss of innate immunity to MTB in diabetic condition and these delays make DM patients at high risk of TB [18]. Moreover, not only innate but adaptive immunity is also affected in the DM more than the normal condition. In many studies the altered level of several cytokines such as IFN-*γ*, IL-12, IL-2, and TNF-*α* was observed in DM-TB conditions.

### 2.3. Drug-Drug Interaction

Anti-TB drug rifampicin is a strong hepatic enzyme-inducer. It boosts the metabolism of various oral hypoglycemic factors, primarily sulphonylureas and biguanides, and also decreases their level in plasma leading to hyperglycemia in diabetic patients. In nondiabetics, it increases the intestinal absorption of glucose and may mimic the symptoms of diabetes [19]. Additionally, some of the anti-TB drugs (particularly rifampicin) make the situation sometime worsen to control DM. This kind of problem arises when anti-TB and DM medication, that is, ATT drug rifampicin, interact with each other. Some concept has been emerged that DM medicines can reduce the impact of anti-TB drug [20, 21]. INH, unlike Rif, inhibits the metabolism of oral hypoglycaemic factors which increases the plasma levels of these drugs. INH mainly interacts with sulphonylureas and leads to diminish control of medicines on DM by antagonizing the effect [22]. It also impairs the release of insulin and causes hyperglycemia even in nondiabetics.

## 3. Consequences of DM on Clinical Characteristics of TB

### 3.1. Age

Data available till now proves that DM-TB patients are of older age than those who do not suffer from DM; the reason may be because DM type 2 persists more in old age people [23].

### 3.2. Gender

It is still controversial that DM-TB is gender specific or not; however, some reports show it is indeed gender specific and persists more in men than women [24, 25].

### 3.3. Symptoms

Symptoms of DM and TB are weight loss and fatigue which are common for both diseases [21]. However, some literature states that body weight of DM-TB patient is usually more than the TB patients not affected by diabetes [12, 23].

### 3.4. Pulmonary/Extrapulmonary

Involvement of extrapulmonary cases has been reported to be less common among diabetic TB patients than in nondiabetics and found more in pulmonary cases [23].

### 3.5. Hemoglobin Level

HbA1C is an abbreviation of hemoglobin A1c that binds to glucose which is also known as glycohemoglobin. In DM-TB patients HbA1C is found to be higher than in patients with DM without TB infection; however few reports suggest that there is no such difference in DM-TB with or without TB [12, 26].

### 3.6. Diagnosis

Similarly some conflicts have been also reported regarding the negative and positive sputum smears in DM-TB [12]. Some reports suggest DM as an independent risk factor for numerous acid fast bacilli on the sputum smear examination [25] and others showed no association between DM and patients [27]. Conflict in these clinical characteristics might be due to the availability of limited studies on status of DM-TB twin burden (Table 1).

## 4. DM Enhances Susceptibility to TB Infection

As already discussed, DM is a global epidemic disease affecting both the developed and developing countries. Due to the immunosuppressed conditions developed in diabetic condition, it makes the individual become more susceptible to MTB infection and to several other diseases linked with immunocompromised condition leading to treatment failure and death. The immense rate of TB with increasing DM has been studied extensively but the complete mechanistic rationale for how DM is making TB more susceptible is still comparatively less understood. The immune response upon the invasion of MTB in the host is well studied through various workers.

**Figure 4:**
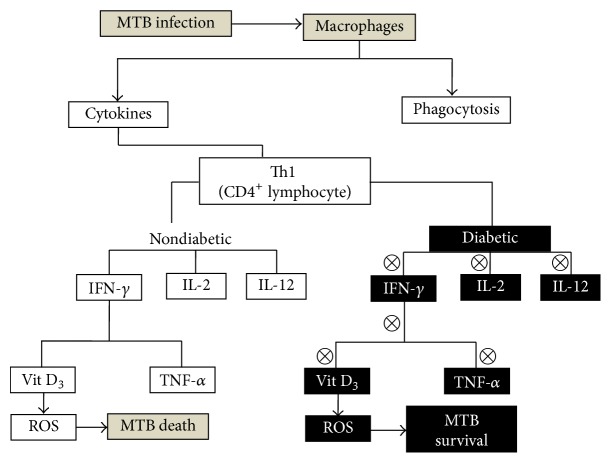
Mechanisms involved in enhancing TB infection during DM: during MTB infection the host innate immunity activates macrophages which causes phagocytosis and activates cytokines. Under nondiabetic condition cytokines become activated leading to ROS generation subsequently killing MTB. Under diabetic condition due to immunocompromised state the alteration in cytokine release leads to the survival of MTB due to suppressed ROS generation.

Inflammatory responses in the host against MTB begin with the first-line defense through nonspecific mechanistic action of macrophages such as phagocytosis and cytokines production (Figure 4). On recognizing the MTB epitope on macrophages, Th1 cytokines like IFN-*γ* and IL-2 are produced by specific CD4^+^ lymphocytes [28]. The functioning of macrophages is enhanced by IFN-*γ* killing or suppressing the replication of phagosome residing organism [29]. IFN-*γ* arouses the TNF and VitD_3_ production that suppresses the growth of MTB in human macrophages through ROS generation, nitrogen intermediate, and alteration in intracellular iron level [30]. IL2 functions by promoting the proliferation of activated lymphocytes. It is seen that if any mutation in IFN-*γ*, IL-12 production, and IL-12 receptor happens that leads to inactivation or nonfunctioning of these cytokines it results in enhanced susceptibility to MTB. If IFN-*γ* in particular has become inactive or nonfunctional particularly in DM then MTB attack is more rigorous (Figure 4) [31]. Several T-lymphocytes are involved in the inflammatory response against MTB such as CD-1 restricted T cells, CD4^+^ T cells, CD8^+^ T cells, and *γ*/*δ*. Among all, CD4^+^ T cells and CD8^+^ T cells play a critical role in immune response against MTB [32, 33]. It has been shown that, in comparison to healthy individuals, the patients with mild, moderate, or advanced pulmonary disease were having enhanced levels of IFN-*γ*, IL-2, IL-4, and IL-10. Further it was observed that IFN-*γ* and IL-2 were higher in mild and moderate patients compared with the patients suffering from advanced disease. However, the patients with moderate and advanced pulmonary disease were having enhanced levels of cytokines IL4 and IL-10 in contrast to mild disease patients [34]. Apart from IFN-*γ* and IL-2, the in vitro studies of relation with the signaling pattern of these cytokines with some other cytokines which are assumed to be involved in the pathogenesis of MTB were also studied, such as TGF-*β* and IL-1*β* [35].

The immune responses in DM cases showed that alterations in humoral innate immune responses are mediated through change in cytokine levels (Figure 4). Deeper studies reflected the declination in the functioning of DM affected macrophages and polymorphonuclear cells through affected chemotaxis, phagocytosis, and killing efficiency [36]. Evidences show the alteration in TNF-*α* and IL-6 level without any stimuli in DM type 1 and DM type 2, respectively. However, under similar condition IL-8 concentration was altered in both DM type 1 and DM type 2 [37, 38]. In the presence of stimuli, such as LPS, the IL-1 secretion of PBMC was found to be downregulated in both type 1 and 2 diabetic PBMC but no difference was found in the level of TNF-*α* [39]. Literatures also suggest that IL-1 and IL-6 production were found to be lowered after LPS stimulation in monocytes of DM type 1 when compared with DM type 2 and control patients, but again no difference was found in TNF-*α* level. Moreover no effect of glucose or insulin stimulation was observed on either of these cytokines which substantially points out that defective LPS may be the important property of diabetic cells [40].

Although the immunologic responses in TB and DM conditions are extensively studied, the responses against MTB in diabetic condition are still not completely understood. However in recent study it was shown that the intracellular level of cytokines, such as IFN-*γ*, IL-2, TNF-*α*, and IL-17, is found to be diminished in the patients with DM or pre-DM. Further it was seen that this diminished level of cytokines is associated with proinflammatory cytokines such as IL-1*β* and IL-18 which were also found to be diminished; however no links were found with the diminished level of type 2 cytokines. Moreover, patient suffering from DM was found to have inhibited level of MTB antigen specific cytokines (type 1 and type 17) [41]. Elevation in resistin level, a protein produced by immune cells in humans, diminishes the activation of mycobacterium-induced inflammasome through suppressing ROS production in leukocytes [42]. In some studies Vitamin D is proposed as an adjuvant for MTB treatment. It was seen that the expression of TLR-2/1 receptor and CYP27B1-hydroxylase enzyme increases upon stimulation and catalyzes the conversion of Vitamin D from its active form to induce antimicrobial peptide cathelicidin finally leading to the MTB execution. Further investigation reported the expression level of CYP27B1-hydroxylase enzyme, VDR, and antimicrobial peptides gene and compared the DM type 2 and TB patients with healthy control. They found that DM type 2 individuals were having lower VDR and antimicrobial peptides expression, however when MDM isolated from DM type 2 patients having low VDR expression upon Vitamin D supplementation were efficiently inhibiting the MTB functioning [43]. GSH level was downregulated in DM type 2 individuals through hyperactivation of transforming growth factor-*β* (TGF-*β*), a cytokine responsible for lowering the GCLC expression. This suggests that the lower level of GSH in DM type 2 individuals makes MTB more susceptible. In DM individuals, level of cytokines such as IL-6 and IL-17 was also found to be increased leading to the enhanced free radicals formation which confirms the degraded level of GSH in DM type 2 individuals. However, it was observed that the increment in the GSH level advances the action against MTB infections. Moreover, cytokines accountable for MTB infection at cellular level, such as TNF-*α*, IL-1*β*, IL-2, IFN-*γ*, and IL-12, were found to be altered in DM type 2 individuals; interestingly the augmented level of some immunosuppressive cytokines such as IL-10 was also found in the same individual [44]. Even short chain fatty acid could also be responsible for the enhanced susceptibility of MTB where the effect of butyrate on different immunologic condition of cytokines in PBMC of diabetic individuals was studied. It was found that the MTB induced proinflammatory cytokine responses were decreased by butyrate treatment in DM individuals; however IL-10 concentration was increased [45]. These links of immunologic condition with DM and MTB suggest that the DM provokes the MTB by providing a conducive environment for its pathogenesis.

## 5. Preventive Measures to Combat DM-TB

Controlling the level of glucose probably minimizes the chances of developing TB among DM people; however sufficient research is still to be conducted which might give awareness whether it is minimizing the TB risk or saving people from death. Gliptins, a new agent for the treatment of type 2 DM also known as DPP-4 inhibitors (inhibitors of dipeptidyl peptidase 4), control the glucagon release and stimulate the release of insulin which lowers the blood glucose level [45]. Therefore, the dose of insulin should be modified while prescribing these drugs to patients [46]. Furthermore, till now how to standardize the control of glucose level in people who are suffering from both TB and DM is not clear. It has been recommended to perform tuberculin skin test (TST) in all the diabetic patients by the American Thoracic Society with purified protein derivative (PPD). If the skin becomes firm reddish bump and is found to be more than 10 mm, then it is suggested to take precautionary treatment with isoniazid (INH) for 6 to 12 months; however, some investigators have challenged the authenticity of this recommendation. Consequently, the persistence of TB infection in DM is high and the sensitiveness of TST may be low [26]. Few studies have explained the benefit of TB prophylaxis in DM patients. The first one was done in Germany in the 1950s where they evaluated the recurrence rate of TB and found it to be lower in DM patients where the treatment has been completed with INH [47]. Second study was done in Russia in the 1960s, where administration of INH analogue in DM patient decreases the occurrence of TB 2-3-fold [48]. Both studies were controversial because of lack of sufficient data regarding interventions [49]. Therefore, only through a randomized controlled trial can the true effectiveness be properly addressed. To attain success in TB-DM disease consequently it needs a cooperate response from each level of the health system, that is, from the administration of public policies to the management of health disease control programs. Policies maker may take steps for establishing new policies and new global health services. Agenda can be focused on combating against this serious threat before it causes more damage to human health as well as economy. Research community synergistically with clinicians and policy makers is required to work in tandem to bring forth some potential measure for the management of the twin burden of TB-DM.

## 6. Conclusion

Patients suffering from DM are more prone to develop TB as it compromises treatment of TB in diabetic patients. Moreover, DM is associated with enhanced risk of multiple outcomes of not only treatment failure but relapse and death during TB treatment. Therefore, extensive studies on TB management with DM are required. Considering the increasing disease burden of DM, particularly in highly prevalent TB areas, TB control programs will need to expand their efforts and focus on treatment of patients with DM and TB.

## Figures and Tables

**Table 1:** Showing differences in clinical characteristics between TB and DM-TB.

Characteristics	TB	DM-TB
Age	Not defined and persisting at any age	Usually persisting in adults and in old age
Gender	Men or women	Persisting more in men
Symptoms	Higher body weight loss, less fatigue, low breathlessness, less hemoptysis, more fever, more expectoration	Lesser body weight loss, more fatigue, higher breathlessness, more hemoptysis, less fever, lower expectoration
Pulmonary/ extrapulmonary	Both pulmonary and extrapulmonary involvement	Only pulmonary and few extrapulmonary involvement
Hemoglobin level	Anemia	HbA1C is higher
Diagnosis	Can be diagnosed	Difficulty in diagnosis

## Abbreviations

TB: Tuberculosis
DM: Diabetes Mellitus
AIDS: Acquired immune deficiency syndrome
HIV: Human immunodeficiency virus
MTB: * Mycobacterium tuberculosis*
PPD: Purified protein derivative
Anti-TB: Antituberculosis
ATT: Antituberculosis therapy
INH: Isoniazid
Rif: Rifampicin
TST: Tuberculin skin test
IFN-*γ*: Interferon-gamma
IL-2: Interleukin-2
TNF: Tumor necrosis factor
VitD3: Vitamin D3
TGF-*β*: Transforming growth factor-*β*
LPS: Lipopolysaccharide
PBMC: Peripheral blood mononuclear cells
ROS: Reactive oxygen species
VDR: Vitamin D receptor
MDM: Monocytes derived from macrophages
GSH: Glutathione
GCLC: Glutamine-cysteine ligase.
